# Acute kidney injury prevalence, progression and long-term outcomes in critically ill patients with COVID-19: a cohort study

**DOI:** 10.1186/s13613-021-00914-5

**Published:** 2021-08-06

**Authors:** Nuttha Lumlertgul, Leah Pirondini, Enya Cooney, Waisun Kok, John Gregson, Luigi Camporota, Katie Lane, Richard Leach, Marlies Ostermann

**Affiliations:** 1 Department of Critical Care, Guy’s & St Thomas’ Hospital NHS Foundation Hospital, 249 Westminster Bridge Road, London, SE1 7EH UK; 2 Division of Nephrology and Excellence Centre for Critical Care Nephrology, King Chulalongkorn Memorial Hospital, Bangkok, Thailand; 3 Critical Care Nephrology Research Unit, Chulalongkorn University, Bangkok, Thailand; 4 Department of Medical Statistics, London School of Hygiene and Tropical Medicine, London, UK

**Keywords:** COVID-19, SARS-CoV-2, Acute kidney injury, Kidney replacement therapy, Recovery, Dialysis, AKI

## Abstract

**Background:**

There are limited data on acute kidney injury (AKI) progression and long-term outcomes in critically ill patients with coronavirus disease-19 (COVID-19). We aimed to describe the prevalence and risk factors for development of AKI, its subsequent clinical course and AKI progression, as well as renal recovery or dialysis dependence and survival in this group of patients.

**Methods:**

This was a retrospective observational study in an expanded tertiary care intensive care unit in London, United Kingdom. Critically ill patients admitted to ICU between 1st March 2020 and 31st July 2020 with confirmed SARS-COV2 infection were included. Analysis of baseline characteristics, organ support, COVID-19 associated therapies and their association with mortality and outcomes at 90 days was performed.

**Results:**

Of 313 patients (70% male, mean age 54.5 ± 13.9 years), 240 (76.7%) developed AKI within 14 days after ICU admission: 63 (20.1%) stage 1, 41 (13.1%) stage 2, 136 (43.5%) stage 3. 113 (36.1%) patients presented with AKI on ICU admission. Progression to AKI stage 2/3 occurred in 36%. Risk factors for AKI progression were mechanical ventilation [HR (hazard ratio) 4.11; 95% confidence interval (CI) 1.61–10.49] and positive fluid balance [HR 1.21 (95% CI 1.11–1.31)], while steroid therapy was associated with a reduction in AKI progression (HR 0.73 [95% CI 0.55–0.97]). Kidney replacement therapy (KRT) was initiated in 31.9%. AKI patients had a higher 90-day mortality than non-AKI patients (34% vs. 14%; *p* < 0.001). Dialysis dependence was 5% at hospital discharge and 4% at 90 days. Renal recovery was identified in 81.6% of survivors at discharge and in 90.9% at 90 days. At 3 months, 16% of all AKI survivors had chronic kidney disease (CKD); among those without renal recovery, the CKD incidence was 44%.

**Conclusions:**

During the first COVID-19 wave, AKI was highly prevalent among severely ill COVID-19 patients with a third progressing to severe AKI requiring KRT. The risk of developing CKD was high. This study identifies factors modifying AKI progression, including a potentially protective effect of steroid therapy. Recognition of risk factors and monitoring of renal function post-discharge might help guide future practice and follow-up management strategies.

*Trial registration* NCT04445259

**Supplementary Information:**

The online version contains supplementary material available at 10.1186/s13613-021-00914-5.

## Background

Acute kidney injury (AKI) is prevalent in coronavirus disease-19 (COVID-19) patients. The pathogenesis is multifactorial including possible viral invasion, hypovolaemia, systemic inflammation, nephrotoxin exposure, endothelial dysfunction, coagulopathy, and organ crosstalk [1, 2]. The incidence of AKI varies among geographical regions and clinical settings, ranging from 7 to 57% in hospitalised patients [3–5] and 19–80% in patients in the intensive care unit (ICU) [6–10]. Kidney replacement therapy (KRT) is utilised in 20–60% [4, 11]. Both AKI and KRT are associated with adverse hospital outcomes and increased mortality [12].

Risk factors for AKI development include increased age, comorbidities, and racial background [13]. However, only few studies have examined the impact of COVID related therapies on risk of AKI progression [14] or long-term renal outcomes beyond hospital discharge in critically ill patients with COVID-19.

During the first wave of the COVID-19 pandemic, more than 10,000 patients were admitted to critical care in the United Kingdom (UK) [15]. Critical care bed capacity surged from 7.6 to 19.6 beds per 100,000 population [16]. The high prevalence of AKI and limited supply of consumables led to significant challenges and disruptions in KRT delivery [17, 18].

Understanding the burden of short- and longer-term complications is critical for prognostication, clinical management, and future resource planning. The aims of this study were to assess the AKI prevalence, trajectories, impact of COVID related therapies and longer-term renal outcomes following severe COVID-19.

## Materials and methods

### Setting and Study design

We retrospectively analysed all patients admitted to the ICU in a UK tertiary-level critical care unit during the first COVID wave at a time when critical care bed capacity had expanded from 64 to 160 beds.

### Participants

All adult patients (≥ 18 years old) who were admitted to ICU between 1st March and 31st July 2020 with a clinical diagnosis of COVID-19 following a confirmatory reverse transcriptase-polymerase chain reaction (RT-PCR) test of nasopharyngeal or endotracheal samples were included. We excluded (1) patients with pre-existing end-stage kidney disease; (2) kidney transplant recipients, and (3) patients in whom COVID-19 was not the cause of ICU admission. If there were multiple admissions, only the first admission to ICU was included.

### Data collection

Patient-level data (Additional file 1: Methods) were collected from the electronic health records through a combination of manual review by two independent trained data collectors and automated laboratory parameter extraction. Any disagreement was adjudicated by a third independent investigator.

### Outcomes

The primary outcome was the incidence of AKI within 14 days after ICU admission. AKI was diagnosed and staged according to the Kidney Disease Improving Global Outcomes (KDIGO) criteria [19]. In brief, AKI is defined by an absolute increase in serum creatinine (SCr) by 0.3 mg/dL (26.4 µmol/L) within 48 h of ICU admission, an increase in SCr ≥ 1.5 times from baseline within 7 days, or urine output < 0.5 mL/kg/h for ≥ 6 h. Both, SCr and urine output criteria were employed. Adjusted ideal body weight was used to calculate urine output per hour in obese patients [20]. Baseline SCr was determined from outpatient SCr values between 7 and 365 days before ICU admission. If a historical SCr result was not available, the first SCr on hospital admission [11] was used or SCr was determined by back-calculation using the Modification of Diet in Renal Disease (MDRD) formula and an assumed estimated glomerular filtration rate (eGFR) of 75 mL/min/1.73 m^2^ [19]. Baseline AKI stage on admission and maximal AKI stage were reported. “AKI at ICU admission” was defined as any stage AKI present within 48 h of ICU admission [21]. AKI progression was defined as either new or worsening AKI to stage 2/3 after 48 h.

Secondary outcomes were KRT use during ICU stay, hospital mortality, 90-day mortality, dialysis dependence at hospital discharge and at 90 days, renal recovery at discharge and 90 days, and major adverse kidney events at 90 days (MAKE90). Renal recovery was defined as having SCr < 1.5 times of baseline and being dialysis independent at hospital discharge or at 90 days [22]. The composite outcome MAKE90 comprises of non-renal recovery, dialysis dependence, or death. SCr at 90 days was sought from the medical health records; if not available, the patient's general practitioner (GP) was contacted.

### Statistical analysis

Baseline characteristics were summarised by the final AKI diagnosis. Baseline laboratory measurements used are the first values recorded within 24 h of ICU admission. Missing values were imputed using the most common category for categorical variables and the median value for continuous variables (Table 1). We performed 3 main analyses. First, we used logistic regression and forward stepwise selection (p-value threshold 0.05) to identify variables associated with AKI at ICU admission. We included the variables given in Table 1 as candidate covariates, except for variables included in the definition of AKI (SCr, urine output) and variables representing frailty indices (APACHE II, Charlson Comorbidity Index (CCI) and Clinical Frailty Score (CFS)). Second, we examined risk factors for progression to stage 2/3 AKI after ICU admission. For this, we excluded patients with AKI stage 2/3 diagnosed within 48 h from ICU admission. Cox proportional hazards models with forward stepwise selection (*p*-value threshold 0.05) was used. Hazard ratios (HRs) for ICU-related treatments or complications adjusted for baseline predictors of AKI progression were calculated. We used time-updated covariates to model ICU-related treatments and complications. Patients were censored at whichever of the following occurred first: AKI progression, death, or 90-day from ICU admission. Six patients were censored between 74 and 87 days due to database lock. Finally, we compared 90-day mortality according to maximal AKI stage using Kaplan–Meier curves. We calculated HRs for mortality according to time-updated maximal AKI stage. We additionally examined predictors of 90-day mortality using the same methods as applied to examine predictors of AKI progression. Analyses were conducted with R version 3.6.1.

**Table 1:** Baseline characteristics of patients and final acute kidney injury status

Characteristics	Overall n = 313^a^	No AKI n = 73^a^	AKI n = 240^a^
Age (years)	54.5 (13.9)	51.6 (13.8)	55.4 (13.9)
Male sex	219 (70%)	49 (67%)	170 (71%)
Ethnicity: White	119 (46%)	25 (42%)	94 (47%)
Black	90 (35%)	16 (27%)	74 (37%)
Others	49 (19%)	18 (31%)	31 (16%)
Admission: ED	94 (30%)	18 (25%)	76 (32%)
Ward	102 (33%)	24 (33%)	78 (32%)
Transfer from other ICUs	114 (36%)	31 (42%)	83 (35%)
Others	3 (1.0%)	0 (0%)	3 (1.2%)
Infection setting: Community-acquired	275 (90%)	61 (85%)	214 (91%)
Hospital acquired	8 (2.6%)	1 (1.4%)	7 (3.0%)
Occupational	24 (7.8%)	10 (14%)	14 (6.0%)
BMI (kg/m^2^)	29.6 (6.5)	27.9 (5.2)	30.1 (6.7)
Current smoker	13 (6.1%)	6 (11%)	7 (4.3%)
Admission SOFA score	5.4 (2.6)	3.9 (2.3)	5.9 (2.5)
APACHE II Score	14.2 (4.8)	12.5 (4.8)	14.7 (4.7)
Clinical frailty score	2.6 (1.0)	2.4 (0.9)	2.6 (1.0)
Comorbidities: diabetes	95 (30%)	12 (16%)	83 (35%)
Asthma	48 (15%)	14 (19%)	34 (14%)
Hypertension	126 (40%)	19 (26%)	107 (45%)
Coronary artery disease	15 (4.8%)	2 (2.7%)	13 (5.4%)
Congestive heart failure	14 (4.5%)	2 (2.7%)	12 (5.0%)
Atrial fibrillation/atrial flutter	11 (3.5%)	1 (1.4%)	10 (4.2%)
COPD	12 (3.8%)	0 (0%)	12 (5.0%)
Chronic kidney disease	22 (7.0%)	1 (1.4%)	21 (8.8%)
Chronic liver disease	12 (3.8%)	1 (1.4%)	11 (4.6%)
HIV infection	7 (2.2%)	3 (4.1%)	4 (1.7%)
Malignancy	14 (4.5%)	3 (4.1%)	11 (4.6%)
Other coinfections	42 (13%)	9 (12%)	33 (14%)
Charlson Comorbidity Index	0.8 (1.3)	0.6 (1.2)	0.9 (1.3)
Type of ventilation on admission:			
Invasive	254 (81.2%)	48 (65.8%)	206 (85.8%)
Non-invasive	4 (1.3%)	0	4 (1.7%)
High-flow nasal cannula	21 (6.7%)	8 (11.0%)	13 (5.4%)
None	34 (10.9%)	17 (23.3%)	17 (7.1%)
Vasopressor support on admission	133 (42.5%)	23 (31.5%)	110 (45.8%)
ECMO	58 (18.5%)	18 (24.7%)	40 (16.7%)
Medications: ACE-Inhibitor	54 (17%)	8 (11%)	46 (19%)
ARB	39 (12%)	5 (6.8%)	34 (14%)
Baseline creatinine^b^	86.2 (38.7)	73.7 (31.7)	90.0 (39.9)
pH	7.4 (0.1)	7.4 (0.1)	7.4 (0.1)
PaO_2_ (kPa)	11.6 (5.0)	11.7 (5.1)	11.5 (5.0)
Ionised calcium (mmol/L)	1.1 (0.1)	1.1 (0.1)	1.1 (0.1)
Lactate (mmol/L)	2.2 (2.0)	1.9 (1.8)	2.2 (2.0)
Chloride (mEq/L)	99.8 (5.7)	99.5 (6.4)	99.9 (5.5)
White blood cells (10^9/L)	9.9 (4.8)	9.7 (5.2)	9.9 (4.6)
Neutrophils (10^9/L)	8.4 (4.4)	8.4 (4.7)	8.4 (4.3)
Lymphocytes (10^9/L)	0.8 (0.5)	0.9 (0.4)	0.8 (0.6)
Haemoglobin (g/L)	115.8 (22.1)	114.4 (24.5)	116.2 (21.3)
Platelet (10^9/L)	253.2 (108.7)	284.1 (128.8)	243.8 (100.3)
Urea (mmol/L)	10.1 (9.4)	6.7 (4.3)	11.0 (10.2)
Creatinine (µmol/L)	121.0 (101.7)	70.4 (30.7)	136.4 (110.4)
Albumin (g/L)	31.2 (6.4)	31.1 (6.3)	31.2 (6.4)
ALT (U/L)	67.2 (115.7)	49.8 (41.9)	72.4 (129.2)
Bilirubin (µmol/L)	12.8 (16.3)	9.4 (5.8)	13.7 (18.2)
CRP (mg/L)	203.6 (137.3)	166.9 (129.7)	214.9 (137.8)
Sodium (mmol/L)	138.5 (6.3)	139.3 (6.6)	138.3 (6.2)
Potassium (mmol/L)	4.5 (0.8)	4.3 (0.7)	4.6 (0.9)
Bicarbonate (mmol/L)	24.5 (5.5)	26.9 (6.6)	23.8 (5.0)
Fibrinogen (g/L)	6.8 (2.0)	6.7 (1.9)	6.8 (2.1)
Ferritin (ng/mL)	1,991.3 (2,672.8)	1,332.2 (1,233.7)	2,199.1 (2,958.3)
PaO_2_/FiO_2_, kPa	21.0 (12.5)	23.3 (11.9)	20.4 (12.6)
Urine output (ml)	1,078.0 (818.5)	1,289.9 (787.0)	1,014.1 (818.6)

^a^Statistics presented: mean (SD); *n* (%)

^b^Determined at true baseline creatinine (*n* = 106), first hospital admission (*n* = 130) or baseline creatinine back calculated by MDRD formula (*n* = 77)

Missing: ethnicity (*n* = 55), infection setting (*n* = 6), smoker (*n* = 99), homeless (*n* = 10), BMI (*n* = 33)

Missing: pH (*n* = 1), PaO_2_ (*n* = 8), ionised calcium (*n* = 3), lactate (*n* = 1), chloride (*n* = 6), white blood cells (*n* = 5), neutrophils (*n* = 24), lymphocytes (*n* = 5), Hb (*n* = 5), platelet (*n* = 5), urea (*n* = 17), creatinine (*n* = 4), albumin (*n* = 4), ALT (*n* = 9), bilirubin (*n* = 10), CRP (*n* = 7), sodium (*n* = 3), potassium (*n* = 4), bicarbonate (*n* = 3), fibrinogen (*n* = 35), ferritin (*n* = 46), PaO_2_/FiO_2_ ratio (*n* = 9), urine output (*n* = 2)

*COPD* chronic obstructive pulmonary disease, *ED* emergency department, *HIV* human immunodeficiency virus, *SOFA* Sequential Organ Failure Assessment, *APACHE II* Acute Physiologic and Chronic Health Evaluation II, *ACE* angiotensin-converting enzyme, *ARB* angiotensin receptor blocker, *ALT* alanine transaminase, *CRP* C-reactive protein, *ECMO* extracorporeal membrane oxygenation, *BMI* body mass index, *kPa* kiloPascal

## Results

Between 1st March and 31st July 2020, 335 critically ill COVID-19 positive patients were admitted to Critical Care (Additional file 1: Figure S1) of whom 313 patients were included in the final analysis [70% male, mean age 54.5 standard deviation (SD) 13.9 years]. The mean APACHE II score and SOFA score were 14.2 ± 4.8 and 5.4 ± 2.6, respectively; 81.2% patients received invasive mechanical ventilation, 42.5% patients received vasopressor support, and 18.5% received veno-venous ECMO on admission. Baseline characteristics and laboratory data by final AKI diagnosis are shown in Table 1.

### AKI diagnosis and staging

AKI was present in 240 (76.7%) patients throughout ICU stay of whom 20.1% had AKI stage 1, 13.1% stage 2, and 43.5% stage 3 (Additional file 1: Table S1). SCr criteria alone accounted for 22.5%, urine output criteria alone for 16.3%, and both criteria defined 61.3% of patients as having AKI. True baseline SCr was available for 35.8% patients.

### AKI at ICU admission

Among all AKI patients, 76% had AKI within 48 h of ICU admission. Risk factors included age, higher BMI, lower serum bicarbonate, lower platelet count, higher C-reactive protein (CRP), and higher serum lactate (Table 2).

**Table 2:** Baseline predictors of any stage AKI at ICU admission

Characteristic	Odds ratio	95% CI	p-value
Age	1.02	1.00, 1.04	0.024
Female sex	1.09	0.61, 1.96	0.771
BMI (kg/m^2^)	1.05	1.01, 1.10	0.019
Ethnicity			
Not Black	Ref	Ref	
Black	1.66	0.97, 2.88	0.066
Lower baseline HCO_3_^a^	1.07	1.02, 1.13	0.006
Lower baseline platelet^b^	1.34	1.05, 1.73	0.020
Baseline CRP^c^	1.28	1.06, 1.57	0.013
Lower baseline haemoglobin^d^	1.13	1.00, 1.29	0.059
Baseline potassium^e^	1.39	1.00, 1.95	0.051
Baseline lactate^e^	1.31	1.11, 1.61	0.005

^a^ per mEg/L

^b^ per 10^11^/L

^c^ per 100 mg/L

^d^ per 10 g/L

^e^ per mmol/L

*BMI* body mass index, *CRP* C-reactive protein

### AKI progression

115 (36.7%) patients progressed to AKI stage 2/3 after 48 h (Additional file 1: Table S1). Among procedures and complications during ICU stay, invasive mechanical ventilation [adjusted hazard ratios (aHR) 4.11, 95% CI 1.61–10.49] and positive cumulative fluid balance in the first 48 h (aHR 1.21, 95% CI 1.11–1.31), were independent risk factors, whilst new steroid use was associated with a reduced risk of AKI progression [aHR 0.73, 95%CI 0.55–0.97]. Treatment with ECMO, proning, remdesivir and anticoagulation use were not independently associated with AKI progression (Table 3).

**Table 3:** Adjusted* hazard ratios for acute kidney injury progression in intensive care unit according to therapies

Variable	Patients with AKI progression	Patients with no AKI progression	Hazard ratio	95% CI	P-value
Cumulative net balance at 48 h (each 1000 ml)			1.205	1.108, 1.311	< 0.001
Ventilation type					
None	5	26	1 (ref)		
Invasive	89	100	4.114	1.614, 10.485	0.003
Non-invasive/mask/HFNC	6	14	2.677	0.783, 9.150	0.116
Vasopressor					
No	51	94	1 (ref)		
Yes	49	46	1.355	0.906, 2.025	0.139
ECMO					
No	88	112	1 (ref)		
Yes	12	28	0.808	0.411, 1.590	0.538
Prone position					
No	50	100	1 (ref)		
Yes	50	40	0.926	0.671, 1.279	0.642
Remdesivir					
No	98	131	1 (ref)		
Yes	2	9	0.407	0.055, 2.985	0.376
New steroids					
No	28	71	1 (ref)		
Yes	72	69	0.730	0.550, 0.970	0.030
Anticoagulation use (excluding thromboembolism prophylaxis)					
No	47	92	1 (ref)		
Yes	53	48	0.833	0.580, 1.196	0.322
Total	100	140			

^*^adjustment for age, sex, and the following factors measured at ICU admission: AKI stage, HCO_3_^−^, bilirubin, ALT, iCa, PaO_2_/FiO_2_ ratio, BMI, and Charlson Comorbidity Index

HFNC, high-flow nasal cannula; ECMO, extracorporeal membrane oxygenation; AKI, acute kidney injury; CI, confidence interval

### Kidney replacement therapy (KRT)

One-hundred patients (32%) received KRT during ICU stay. Among them, the median time to KRT was 3 days (IQR 1–6) from ICU admission with a median KRT duration of 12 days (IQR 6–22). The initial modality was continuous kidney replacement therapy (CKRT) in 87 patients, prolonged intermittent kidney replacement therapy (PIKRT) in 12 patients, and intermittent haemodialysis (IHD) in 1 patient. The primary indications for KRT were hyperkalaemia (*n* = 37), oliguria (*n* = 67), acidosis (*n* = 25), high urea (*n* = 73), pulmonary oedema (*n* = 12), and others (*n* = 15, including rhabdomyolysis, fluid and electrolyte imbalances).

### ICU outcomes and 90-day mortality

**Fig. 1:**
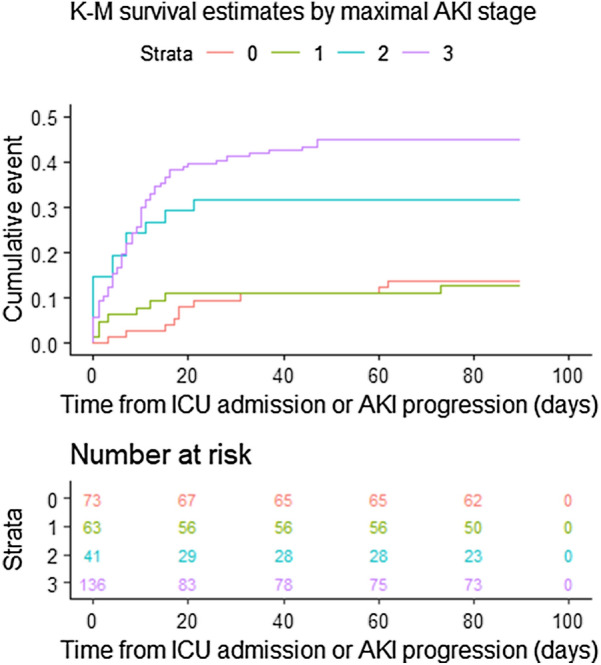
Kaplan–Meier survival curves by maximal acute kidney injury (AKI) stage showing time from intensive care unit admission (in non-AKI patients) or from reaching maximal AKI stage (in AKI patients) to death or censoring at 90 days

Overall ICU mortality of the total cohort was 28%; 33% in AKI patients vs 12% in non-AKI patients, *p* < 0.001. The 90-day mortality was 29%; 34% in AKI versus 14% in non-AKI patients (*p* < 0.001) (Table 4). The HRs for final AKI stage 1, 2, and 3 for 90-day mortality versus no AKI were 2.52 (95% CI 1.11–5.72; *p* = 0.03), 11.1 (95% CI 5.19–23.8; *p* < 0.001), and 5.79 (95% CI 3.49–9.61; *p* < 0.001), respectively. The Kaplan–Meier curves for maximal AKI staging are shown in Fig. 1.

**Table 4:** Key outcomes by final acute kidney injury status

	Overall	Non-AKI	AKI	P value
Outcome	(n = 313)	(n = 73)	(n = 240)	
Kidney replacement therapy use in ICU	100 (32%)	1 (1%)	99 (41%)	< 0.001^a^
90-day mortality^b^	92 (29%)	10 (14%)	82 (34%)	< 0.001^c^
Dialysis dependence at hospital discharge (in survivors)				
No	214 (96%)	64 (100%)	150 (95%)	
Yes	8 (4%)	0 (0%)	8 (5%)	0.109^a^
Dialysis dependence at 90 days (in survivors)				
No	188 (95%)	50 (94%)	138 (96%)	
Yes	9 (5%)	3 (6%)	6 (4%)	0.704^a^
(Missing)	24	10	14	
Renal recovery at discharge^d^	N/A	N/A	129/158 (81.6%)	-
Renal recovery at 90 days^e^	N/A	N/A	130/143 (90.9%)	-

AKI, acute kidney injury; ICU, intensive care unit

^a^ P-value calculated from Fisher’s exact test

^b^ 88 patients died in ICU, 4 patients died outside of ICU (3 in hospital, 1 after hospital discharge)

^c^ P-value calculated from a Cox proportional hazards model using time-updated AKI diagnosis as covariate

^d^ Recovery at discharge is defined as having serum creatinine of < 1.5 times of baseline or being dialysis independent at discharge (within the population of survivors only)

^e^ Recovery at 90 days is defined as having serum creatinine of < 1.5 times of baseline or being dialysis independent at discharge (within the population of survivors only). Note there are 27 missing observations

We identified several factors present on ICU admission that were independently associated with increased 90-day mortality risk: age (HR 1.04; 95% CI 1.03–1.06), male sex (HR 2.17; 95% CI 1.28–3.68), asthma (HR 1.84; 95% CI 1.06–3.19), atrial flutter or fibrillation (HR 2.95; 95% CI 1.10–7.90), high serum lactate (HR 1.25; 95% CI 1.13–1.39), neutrophilia (HR 1.07; 95% CI 1.01–1.13); lower lymphocyte count (HR 2.31; 95% CI 1.32–4.04), lower PaO_2_/FiO_2_ ratio (HR 1.95; 95% CI 1.26–2.98) and lower pH (HR 1.30; 95% CI 1.00–1.69) (Additional file 1: Table S3). With regard to ICU treatments and complications, KRT use, vasopressor support, prone position, and ARDS were independent risk factors for mortality, with aHR 1.69 (95%CI 1.05–2.72), 3.00 (95%CI 1.54–5.86), 1.44 (1.08–1.92), and 2.20 (1.38–3.51), respectively (Additional file 1: Table S4).

### ICU and 90-day renal outcomes and recovery

**Fig. 2:**
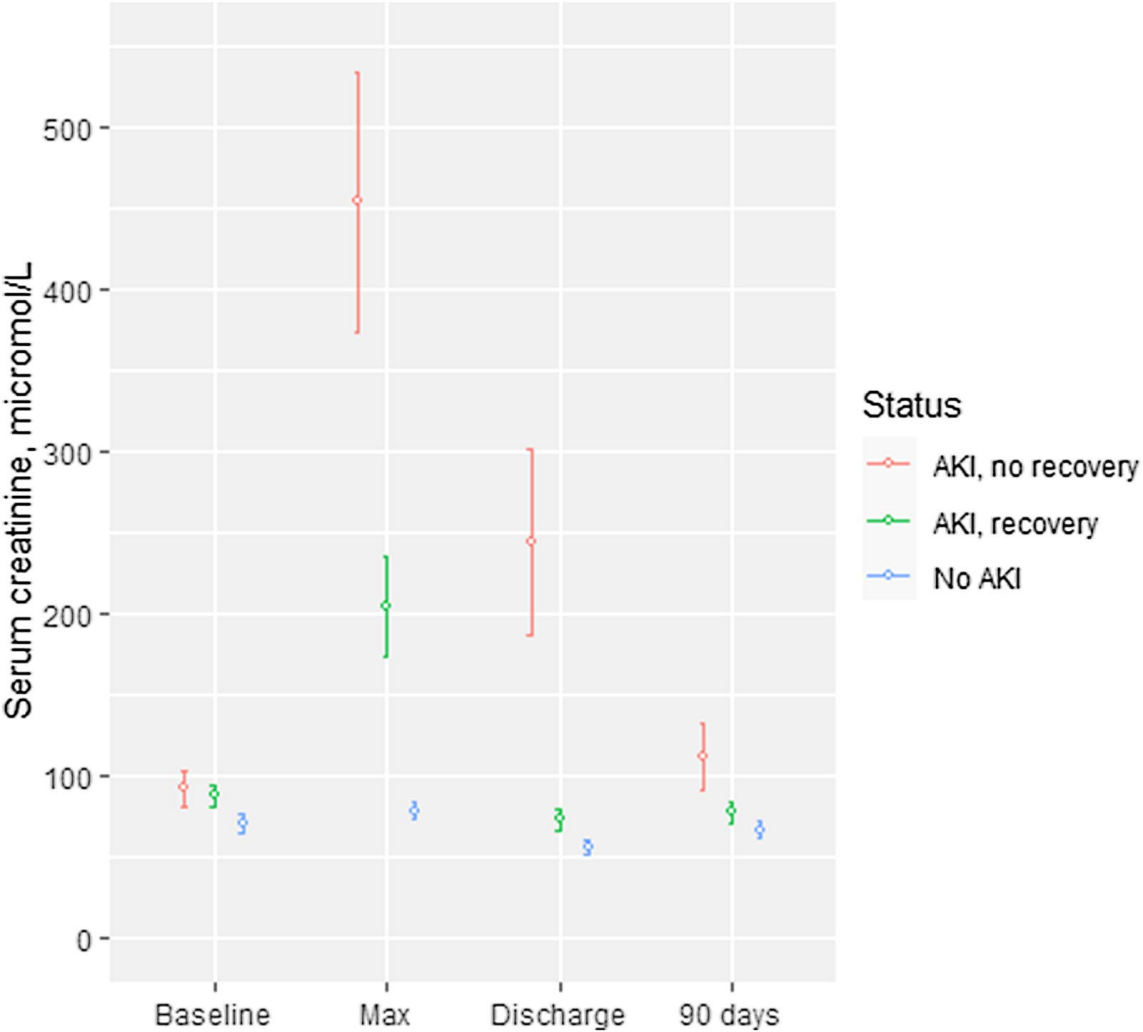
Mean (95% CI) serum creatinine at different time points from baseline to 90 days after hospitalisation in patients who survived, were not dialysis-dependent at hospital discharge, and had serum creatinine measurement available at 90 days (n = 182)

At hospital discharge, 8 of 222 survivors (4%) were dialysis dependent, and 81.6% of survivors had full renal recovery. At 90 days, 9 of 197 survivors (5%) with available data were dialysis dependent. SCr was available in 194 (87.8%) survivors; of whom 90.9% had renal recovery. MAKE90 occurred in 42.4% of all patients. (Additional file 1: Table S5) Of all AKI survivors, 16.5% had CKD (eGFR < 60 ml/min/1.73m2) at 90 days, comprising 44.8% of patients who did not recover renal function at discharge compared with 10.1% of those with renal recovery at discharge, respectively (Additional file 1: Table S6). Figure 2 shows temporal changes in SCr from baseline, maximum, at hospital discharge, and at 90 days in survivors with SCr results available. Compared with baseline SCr, AKI survivors without renal recovery at discharge had higher SCr values at 90 days, while AKI survivors who recovered renal function had lower SCr results; patients without AKI did not have significant changes in SCr post-discharge (Additional file 1: Table S7).

The relationship between final AKI stage and duration is shown in Additional file 1: Table S6. AKI duration was categorised as prolonged (≥ 7 days or non-recovery) in 37.1%, sustained (3–6 days) in 12.8%, and transient (≤ 2 days) in 26.8%, respectively. Prolonged AKI and non-recovery were more frequent in those with final AKI stage 3.

## Discussion

Our data confirm the high prevalence of AKI in critically ill COVID-19 patients during the first wave. Most patients developed AKI stage 3, about one-third of patients received KRT, and the majority of AKI occurred within 48 h of ICU admission. It is the first study which highlights the high risk of progressive AKI after 48 h (36%) in COVID-19 and identifies potentially modifiable risk factors. Although mortality was high in AKI patients, less than 5% of survivors were dialysis dependent at 90 days. However, the considerable risk of CKD at 90 days should be appreciated, especially in patients without renal recovery at hospital discharge.

The high incidence of AKI in our study compared with other studies [6, 10, 23–25] might have several explanations. First, most patients required multi-organ support, including mechanical ventilation (81%), vasopressor support (43%), and ECMO (19%). One-third of patients were referred from other institutions for specialist input or as part of mutual aid during the pandemic. A third were admitted from general medical wards which might reflect AKI onset during hospitalisation before ICU admission. Second, we employed both SCr and urine output criteria to define AKI, where other studies only used SCr results. 16% of cases were identified by urine output criteria alone. Finally, there might have been stricter admission criteria to ICU, given the pandemic and the relatively low number of ICU beds in the UK [7, 26].

Patients presenting with AKI at ICU admission were generally older, had a higher body mass index, and were more acutely ill as evidenced by lower bicarbonate, lower platelet, higher lactate, and higher CRP. As suggested by other studies, this suggests that AKI is a marker of disease severity, and that inflammation might contribute to AKI development [8, 9, 27, 28]. One-third of all patients progressed to moderate or severe AKI after 48 h in ICU. A previous study revealed that 15% of patients had AKI stage ≥ 2 on admission and 60% progressed from stage 2 to 3 or needed KRT afterwards [29]. Similar to non-COVID-19 patients, positive fluid balance in the first 48 h after ICU admission was associated with AKI progression [30–32]. As in previous reports, invasive mechanical ventilation increased the risk of AKI by about fourfold [5, 10, 33–35]. The use of KRT in our cohort was similar to reports from other institutions and national UK data [11, 15], and our association between AKI stage and mortality was also in keeping with other studies [12, 36].

This study included critically ill individuals admitted to critical care during the UK’s first wave of the pandemic. Effective management strategies were unknown during this time. In the following months, randomised controlled trials identified systemic steroid use as an effective COVID-19 therapy [37, 38]. Our results showed for the first time that steroid use was associated with a reduced risk of AKI progression. However, indications for treatment initiation and dosage were not protocolised and left to the clinicians’ discretion at the time. Consequently, indication bias and residual confounding effects cannot be excluded. Also, the exact protective mechanism is not clear from our analysis. We note that renal outcomes were not reported in the RECOVERY trial [38]. At this stage, our results of steroid therapy being protective on renal outcomes should be viewed as hypothesis-generating to guide future adequately powered studies [29].

Proning was associated with higher mortality, which may reflect the severity of COVID-19 rather than the effects of proning on mortality itself [39]. The overall mortality rate was relatively low compared with previous studies comprising patients with similar severity and receiving mechanical ventilation [7, 8, 23, 40]. Possible explanations are younger age and relatively low CCI and CFS. Other reasons may include factors directly related to the pandemic, i.e. changes to ICU organisation, staffing and resource utilisation, and variations in clinical practice that are not captured here.

Renal recovery after AKI is important for patients, families, and all stakeholders involved. Our study shows a 82% recovery rate at hospital discharge based on SCr results and at 90 days with < 5% of patients being dialysis dependent. Other investigations reported renal recovery rates between 17 and 84% and dialysis dependence rates between 8 and 56.5% [11, 18, 41–46]. Our low dialysis dependence rates perhaps may be due to a relatively low proportion of patients with pre-existing CKD. However, loss of muscle mass during critical illness results in a decline in creatinine generation which may lead to an overestimation of renal function as illustrated by our data [47, 48]. Patients with COVID-19 who did not have AKI or had recovered renal function after AKI had a lower SCr at discharge compared to baseline. Our analysis also highlights the significant risk of CKD after COVID-associated AKI: 16% of all AKI survivors had CKD at 3 months. Among those without renal recovery, the incidence was increased to 44%. This is in line with a recent studies showing a fast rate of GFR decline post-AKI [49–51]. If confirmed in future studies, this might pose a significant post-pandemic healthcare and KRT burden which warrants further evaluation [52].

Our study has several strengths. First, two experienced doctors performed detailed chart reviews to collect granular information rather than reliance on administrative data. Second, to our best knowledge, this is the first report where AKI in COVID-19 was defined strictly according to the KDIGO definition using both SCr and urine output criteria. Third, our analysis includes data on AKI prevalence and trajectories, including risk of progression to severe AKI. Fourth, with consent, data were collected from all critically ill patients who were admitted consecutively, reducing selection bias. Fifth, we emphasised on the impact of COVID-specific therapies on AKI risk in the analysis. Steroid use appeared to be protective. A follow-up analysis of data from subsequent waves when steroids were routinely prescribed will be informative. Lastly, we tracked and verified the primary outcome up to 3 months post-discharge, and where necessary, we contacted GPs to obtain SCr results post-discharge. This resulted in 87% availability of follow-up creatinine data and information about the risk of CKD after AKI.

Despite these strengths, we also acknowledge several limitations. First, this was a single-centre observational study which might impact the generalisation of findings. Second, due to the retrospective design, some laboratory results and 90-day SCr results were not available for all patients. We had incomplete information on fluid balance and diuretic use prior to ICU admission, especially since a third of patients were referrals from other institutions. Furthermore, we had no valid data on intravascular fluid status on admission to ICU which is a common problem in critical care settings. Third, urinalysis was not consistently performed in all patients. Fourth, true baseline SCr results were available in only 35%. In the remaining cases, we used the SCr results on admission to hospital or a SCr estimate as the baseline value. Although this is in line with current recommendations [19], we acknowledge that this might have over- or underestimated the true incidence of AKI. Fifth, despite a detailed data collection, we may have missed some relevant nephrotoxic exposures that were not recorded in the medical notes, e.g. use of nephrotoxic drugs at the referring institutions. Sixth, as already mentioned, we judged renal recovery based on SCr results at hospital discharge, but acknowledge that this may have over-estimated kidney function. Although this is a common problem in patients with AKI, we acknowledge that muscle wasting has been reported as a particularly common problem in COVID-19. Lastly, to date we have only follow-up SCr results up to 3 months. Data on kidney function beyond 3 months are not yet available.

## Conclusion

Our analysis confirms a high incidence of AKI and AKI progression in critically ill COVID-19 patients during the first wave. Although 90-day dialysis independence appears to be high, long-term follow-up is needed to plan future crisis resource management and apply CKD progression prevention strategies. Future research should determine the impact of COVID-19-specific treatments on AKI prevention and progression [52].

## Supplementary Information

**Additional file 1.** Additional Methods, Figure S1 and Tables S1–S8

## Data Availability

The datasets used and/or analysed during the current study are available from the corresponding author on reasonable request.

## Abbreviations

aHR: Adjusted hazard ratio
APACHE II: Acute Physiologic and Chronic Health Evaluation II
AKI: Acute kidney injury
CCI: Charlson Comorbidity Index
CFI: Clinical Frailty Scale
CI: Confidence interval
CKD: Chronic kidney disease
COVID-19: Coronavirus disease-19
CKRT: Continuous kidney replacement therapy
CRP: C-reactive protein
ECMO: Extracorporeal membrane oxygenation
HR: Hazard ratio
ICU: Intensive care unit
IHD: Intermittent hemodialysis
IQR: Interquartile range
KDIGO: Kidney Diseases: Improving Global Outcomes
KRT: Kidney replacement therapy
MAKE90: Major adverse kidney events at 90 days
MDRD: Modification of Diet in Renal Disease
PIKRT: Prolonged intermittent kidney replacement therapy
REC: Research Ethics Committee
RT-PCR: Reverse transcriptase-polymerase chain reaction
SCr: Serum creatinine
SD: Standard deviation
SOFA: Sequential Organ Failure Assessment
UK: United Kingdom
