# Supplementary material for: Acute kidney injury prevalence, progression and long-term outcomes in critically ill patients with COVID-19: a cohort study
Source: Ann Intensive Care. 2021 Aug 6;11:123. doi: 10.1186/s13613-021-00914-5 (PMC8343342; doi:10.1186/s13613-021-00914-5)
Supplement: Supplementary file 1 — Additional file 1. Additional Methods, Figure S1 and Tables S1–S8 [file 13613_2021_914_MOESM1_ESM.docx]

**Additional Data**

**Additional Methods**

Baseline characteristics, comorbidities, Charlson Comorbidity Index (CCI), clinical frailty scale (CFS), medications, symptom onset, infection setting, treatment prior to and during hospitalisation, type of respiratory support (i.e. none, oxygen therapy, non-invasive or invasive mechanical ventilation), receipt of extracorporeal membrane oxygenation (ECMO), fluid balance, and laboratory parameters were collected until day 7 of ICU admission, day of discharge from hospital or day of death (whichever occurred first). Additional data were collected on incidence of acute organ failure and use of therapeutic interventions and sequelae until day 14. Disease severity was assessed by Acute Physiologic and Chronic Health Evaluation (APACHE) II score and Sequential Organ Failure Assessment (SOFA) score on admission. Fluid balance was defined by total intake minus total output from 0600am on that day until 0559am on the following day. Cumulative fluid balance at 48 hours was calculated from daily fluid balance on day 1 plus day 2. COVID-related treatments included proning, steroids, antivirals and therapeutic anticoagulation. Complications were adjudicated by the medical team and included acute respiratory distress syndrome (ARDS) defined by the Berlin criteria, congestive heart failure, arrhythmia, cardiac arrest, new infection, new thromboembolic event, new coagulopathy, new major bleeding, and hyperglycaemia.

**Additional Figure S1** **Patient cohort for each analysis stage**


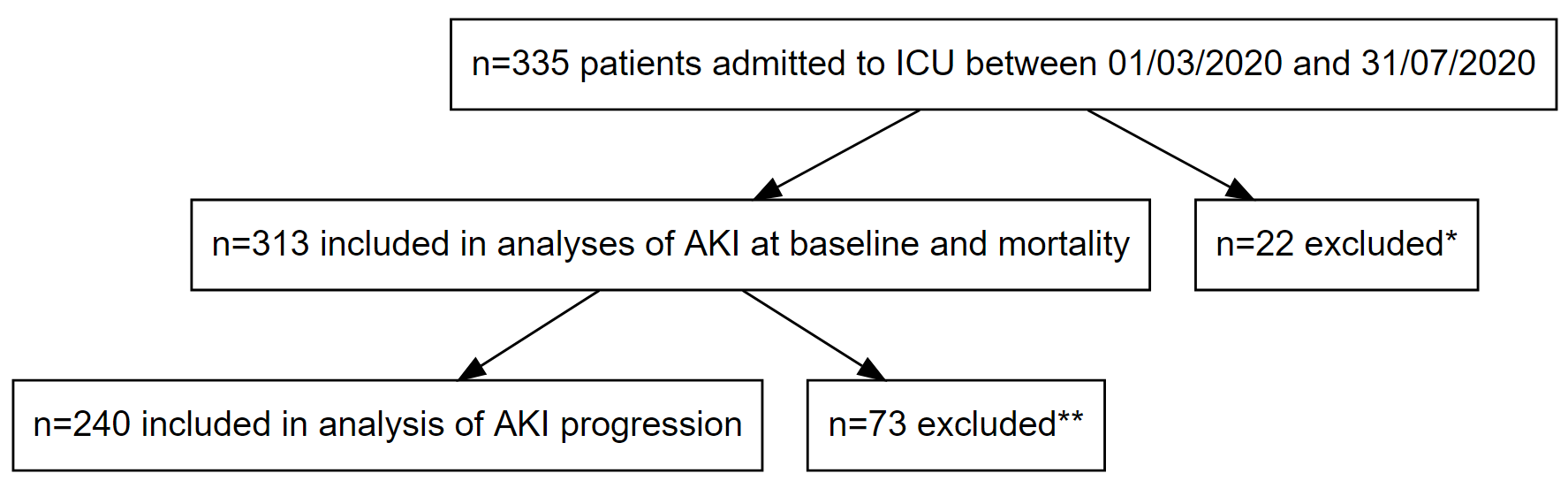


Abbreviations: AKI, acute kidney injury; ICU, intensive care unit

*22 patients were excluded for the following reasons: no positive COVID-19 samples (n=2), end-stage kidney disease (n=7), kidney transplant recipients (n=7), and COVID-19 not primary cause of admission (n=6).

**73 patients were excluded from the analysis of AKI progression for the following reasons: already reached stage 3 AKI by ICU admission (n=58), progression to final stage AKI occurs < 48 hours from ICU admission and hence too early to consider associations with ICU treatments and complications (n=15).

**Additional Table S1** **Acute kidney injury (AKI) stage at intensive care unit admission and final AKI staging.**

| **AKI stage on admission** | **Final AKI Stage** | | | | |
| --- | --- | --- | --- | --- | --- |
|  | **0** | **1** | **2** | **3** | **Total** |
| **0** | 73 | 43 | 25 | 53 | 194 (62%) |
| **1** | 0 | 20 | 12 | 20 | 52 (16.6%) |
| **2** | 0 | 0 | 4 | 5 | 9 (2.9%) |
| **3** | 0 | 0 | 0 | 58 | 58 (18.5%) |
| **Total** | **73 (23.3%)** | **63 (20.1%)** | **41 (13.1%)** | **136 (43.5%)** | **313** |

**Abbreviations:** AKI, acute kidney injury

Yellow boxes indicate no changes between the initial and final AKI stages (n=155),

Orange box indicates progression from AKI stage 0 to 1 after admission (n=43);

Red boxes indicate progression to AKI stage 2/3 after admission (n=115).

**Additional Table S2** **Baseline predictors of acute kidney injury progression**

| **Characteristic** | **Hazard ratio** | **95% CI** | **p-value** |
| --- | --- | --- | --- |
| **Age** | 1.02 | 1.00, 1.03 | 0.012 |
| **Female sex** | 1.23 | 0.76, 1.99 | 0.403 |
| **Baseline AKI stage** |  |  |  |
| 0 | — | — |  |
| 1 | 1.58 | 1.00, 2.49 | 0.050 |
| 2 | 1.34 | 0.49, 3.65 | 0.563 |
| **Lower baseline HCO_3_^-a^** | 1.08 | 1.04, 1.13 | <0.001 |
| **Baseline bilirubin^b^** | 1.71 | 1.17, 2.50 | 0.005 |
| **BMI (kg/m^2^)** | 1.03 | 1.00, 1.07 | 0.067 |
| **Baseline ALT** | 1.43 | 1.10, 1.86 | 0.008 |
| **Baseline iCa^++c^** | 1.29 | 0.99, 1.68 | 0.062 |
| **CCI** |  |  |  |
| CCI 0 | — | — |  |
| CCI 1-2 | 1.61 | 1.04, 2.50 | 0.034 |
| CCI 3-7 | 2.38 | 1.12, 5.05 | 0.024 |
| **Lower log PaO_2_/FiO_2_ ratio** | 1.66 | 1.09, 2.52 | 0.018 |

^a^ per mEq/L lower

^b^ per log µmol/L

^c^ per 100 U/L

^d^ per 0.1mmol/L

**Abbreviations:** AKI, acute kidney injury; BMI, body mass index; CCI, Charlson comorbidity index; ALT, alanine transaminase; iCa, ionised calcium concentration; CI, confidence interval

**Additional Table S3** **Hazard ratios for 90-day mortality adjusted for baseline risk factors**

| **Characteristic** | **Hazard ratio** | **95% CI** | **p-value** |
| --- | --- | --- | --- |
| AKI stage at baseline |  |  |  |
| 0 | — | — |  |
| 1 | 1.38 | 0.81, 2.36 | 0.240 |
| 2-3 | 1.33 | 0.80, 2.22 | 0.273 |
| Age | 1.04 | 1.03, 1.06 | <0.001 |
| Male sex | 2.17 | 1.28, 3.68 | 0.004 |
| Baseline lactate^a^ (per mmol/L) | 1.25 | 1.13, 1.39 | <0.001 |
| Lower baseline lymphocyte count^b^ | 2.31 | 1.32, 4.04 | 0.003 |
| Baseline neutrophil count^c^ | 1.07 | 1.01, 1.13 | 0.022 |
| Lower baseline log PaO_2_/FiO_2_ ratio | 1.94 | 1.26, 2.98 | 0.002 |
| Lower baseline pH^d^ | 1.30 | 1.00, 1.69 | 0.052 |
| Asthma | 1.84 | 1.06, 3.19 | 0.029 |
| AF or AFL | 2.95 | 1.10, 7.90 | 0.031 |

^a^ per mmol/L

^b^ per 10^9^/L lower

^c^ per 10^9^/L

^d^ per 10 units

**Abbreviations:** AKI, acute kidney injury; AF, atrial fibrillation; AFL, atrial flutter

**Additional Table** S**4** **Adjusted* hazard ratios comparing mortality according to intensive care unit (ICU) treatments and ICU complications**

| **Variable** | **Dead** | **Survived** | **Hazard ratio** | **95% CI** | **P-value** |
| --- | --- | --- | --- | --- | --- |
| **Cumulative net fluid balance at 48hr / 1000 mL** |  |  | 1.089 | 0.999, 1.187 | 0.053 |
| **Ventilation Type** |  |  |  |  |  |
| None | 6 | 28 | 1 (ref) |  |  |
| Invasive | 77 | 177 | 2.089 | 0.847, 5.156 | 0.110 |
| BiPAP/CPAP/Mask/HFNC | 9 | 16 | 2.076 | 0.690, 6.249 | 0.194 |
| **KRT use** |  |  |  |  |  |
| No | 49 | 164 | 1 (ref) |  |  |
| Yes | 43 | 57 | 1.691 | 1.051, 2.720 | 0.030 |
| **Vasopressor use** |  |  |  |  |  |
| No | 47 | 133 | 1 (ref) |  |  |
| Yes | 45 | 88 | 3.000 | 1.536, 5.861 | 0.001 |
| **ECMO use** |  |  |  |  |  |
| No | 75 | 179 | 1 (ref) |  |  |
| Yes | 17 | 42 | 1.279 | 0.700, 2.337 | 0.423 |
| **Prone** |  |  |  |  |  |
| No | 38 | 157 | 1 (ref) |  |  |
| Yes | 54 | 64 | 1.437 | 1.076, 1.918 | 0.014 |
| **ARDS^a^** |  |  |  |  |  |
| No | 6 | 71 | 1 (ref) |  |  |
| Yes | 86 | 150 | 2.199 | 1.378, 3.510 | <0.001 |
| **Arrhythmia^b^** |  |  |  |  |  |
| No | 64 | 194 | 1 (ref) |  |  |
| Yes | 28 | 27 | 1.406 | 0.870, 2.271 | 0.164 |
| **New infection^c^** |  |  |  |  |  |
| No | 36 | 103 | 1 (ref) |  |  |
| Yes | 56 | 118 | 1.060 | 0.856, 1.313 | 0.592 |
| **Thrombosis^d^** |  |  |  |  |  |
| No | 65 | 160 | 1 (ref) |  |  |
| Yes | 27 | 61 | 0.967 | 0.604, 1.548 | 0.890 |
| **New major bleeding^e^** |  |  |  |  |  |
| No | 80 | 200 | 1 (ref) |  |  |
| Yes | 12 | 21 | 0.774 | 0.431, 1.389 | 0.391 |
| **Hyperglycaemia^f^** |  |  |  |  |  |
| No | 9 | 48 | 1 (ref) |  |  |
| Yes | 83 | 173 | 1.306 | 0.608, 2.802 | 0.494 |

*each model above is adjusted for all baseline variables presented in Additional table 3

**Abbreviations:** KRT, kidney replacement therapy; ECMO, extracorporeal membrane oxygenation; ARDS, acute respiratory distress syndrome; CI, confidence interval; BIPAP, bilevel positive airway pressure; CPAP, continuous positive airway pressure; HFNC, high flow nasal cannula; ICU, intensive care unit

^a^ Ranieri VM, Rubenfeld GD, Thompson BT, et al. Acute respiratory distress syndrome: the Berlin Definition. *Jama* 2012; 307: 2526-2533.

^b^ Defined as new-onset atrial fibrillation, atrial flutter, ventricular tachycardia, or ventricular fibrillation within 14 days after ICU admission

^c^ Defined as a suspected or confirmed new infection other than COVID-19 that developed after admission to the ICU Should be primarily based on culture data (e.g., sputum, blood, urine, and stool), though other infections can also be included (e.g., cellulitis, abscess) if there was a strong clinical suspicion, even in the absence of positive cultures.

^d^ Defined as new-onset imaging-confirmed deep vein thrombosis, pulmonary thrombosis, thrombotic or embolic stroke, line-related thrombosis, heparin-induced thrombocytopaenia or other thromboembolic events within 14 days after ICU admission

^e^ Defined as bleeding in a critical area or organ (e.g., intracranial, retroperitoneal, pericardial, or intramuscular bleeding with compartment syndrome) or bleeding requiring a procedural intervention (e.g., oesophago-gastro-duodenoscopy or intervention radiology-guided embolisation). Blood transfusion alone does not qualify as an intervention.

^f^ Defined as point-of-care blood sugar ≥10 mmol/l for more than 2 occasions within the same 24 hour-period within 14 days after ICU admission

**Additional Table S5** **Renal recovery status at discharge and at 90 days by final acute kidney injury stage**

| **Survival status** | **At discharge** | | | **At 90 days** | | | |
| --- | --- | --- | --- | --- | --- | --- | --- |
|  | **Survivors** | | **Non-survivors** | **Survivors** | | **Non-survivors** | **MAKE90^c^** |
| **Final AKI Stage** | **Renal function recovered** | **Renal function not recovered^a^** |  | **Renal function recovered** | **Renal function not recovered^b^** |  |  |
| **1** | 54  (41.9%) | 1  (3.4%) | 8  (9.8%) | 48  (36.9%) | 1  (7.7%) | 8  (9.8%) | 9  (9.5%) |
| **2** | 28  (6.2%) | 0 | 13 (15.9%) | 24  (18.5%) | 2  (15.4%) | 13 (15.9%) | 15  (15.8%) |
| **3** | 47  (36.4%) | 28  (96.6%) | 61 (74.4%) | 58  (44.6%) | 10  (76.9%) | 61 (74.4%) | 71  (74.7%) |
| **Total** | 129 (81.6%)^*^ | 29 (18.4%)^*^ | 82 (34.2%)^#^ | 130 (90.9%)^*^ | 13  (9.1%)^*^ | 82 (36.4%)^#^ | 95 (42.4%)^#^ |

**Abbreviations:** AKI, acute kidney injury; MAKE90, major adverse kidney event at 90 days

^a^ Non-recovery at discharge is defined as having creatinine of greater than 1.5 times that at baseline or being dialysis dependent at discharge (within the population of survivors only)

^b^ Non-recovery at 90 days is defined as having creatinine of greater than 1.5 times that at baseline or being dialysis dependent at 90 days (within the population of survivors only). Note there are 15 missing observations.

^c^ MAKE (Major adverse kidney events) comprise non-recovery, dialysis dependence, or death

^*^ Percentage in survivors

^#^ Percentage in all patients

**Additional** **Table S6** **Prevalence of patients with estimated glomerular filtration rate (GFR) < 60 mL/kg/1.73m^2^ at 90 days compared between survivors whose renal function recovered and did not recover.**

|  | **Survivors at 90 days** | | | | | | | | **Non-survivors at 90 days** |
| --- | --- | --- | --- | --- | --- | --- | --- | --- | --- |
| **Final AKI Stage** | **Renal function recovered at discharge** | | | | **Renal function did not recover at discharge** | | | |  |
|  | **eGFR at 90 days (mL/min/1.73m^2^)** | | | | **eGFR at 90 days (mL/min/1.73m^2^)** | | | |  |
|  | **Total** | **< 60** | **≥ 60** | **MISSING** | **Total** | **< 60** | **≥ 60** | **MISSING** |  |
| 1 | 54 | 2 (3.7%) | 45 (83.3%) | 7 (13.0%) | 1 | 0 | 1 (100%) | 0 | 8 |
| 2 | 28 | 5 (17.9%) | 21 (75%) | 2 (7.1%) | 0 | 0 | 0 | 0 | 13 |
| 3 | 47 | 6 (12.8%) | 35 (74.5%) | 6 (12.8%) | 28 | 13 (46.4%) | 12 (42.9%) | 3 (10.7%) | 61 |
| Total | 129 | **13 (10.1%)** | 101 (78.3%) | 15 (11.6%) | 29 | **13 (44.8%)** | 13 (44.8%) | 3 (10.3%) | 82 |

**Abbreviation:** AKI, acute kidney injury; eGFR, estimated glomerular filtration rate

**Additional Table S7 Renal function at baseline, maximum during hospital stay, discharge and 90 days post discharge in patients who survived and were not dialysis dependent at hospital discharge, and had serum creatinine measurement available at 90 days (n=182)**

| **SCr**  **(µmol/L)** | N | SCr at baseline | Maximum SCr | SCr at hospital discharge | At 90 days | | | | |
| --- | --- | --- | --- | --- | --- | --- | --- | --- | --- |
|  |  |  |  |  | SCr level | Change vs. baseline | P-value | Change vs. hospital discharge | P-value |
| **AKI, no recovery*** | 19 | 93 (24.9) | 455 (178.2) | 245 (126.7) | 112 (44.7) | 19.7 | **0.029** | -132.5 | **<0.001** |
| **AKI, recovery** | 114 | 88 (33.9) | 205 (166.4) | 74 (35) | 78 (36.1) | -10.3 | **<0.001** | 4.4 | **0.007** |
| **No AKI** | 49 | 71 (18.6) | 79 (20.3) | 56 (15) | 67 (17.7) | -3.8 | 0.128 | 11.3 | **<0.001** |
| **Overall** | 182 | 84 (30.6) | 197 (177.1) | 87 (73.6) | 79 (35.4) | -5.4 | 0.011 | -8.1 | 0.080 |

Abbreviations: AKI, acute kidney injury; SCr, serum creatinine

values represent mean serum creatinine (SD)

*No recovery is defined as not surviving till hospital discharge, being dialysis dependent at hospital discharge, or having recovery status corresponding to acute kidney disease stages 1, 2, or 3. As we have excluded people who did not survive and who were dialysis dependent at discharge, this group contains only the remaining 19 patients satisfying the third criteria.

**Additional** **Table S8** **Trajectory of AKI by final AKI stage**

|  | **AKI duration^a^** | | | | |
| --- | --- | --- | --- | --- | --- |
| **Final AKI Stage** | **None** | **Transient** | **Sustained** | **Prolonged** | **Total** |
| **0** | 73 (100%) | 0 | 0 | 0 | 73 |
| **1** | 0 | 45 (71.4%) | 9 (14.3%) | 9 (14.3%) | 63 |
| **2** | 0 | 14 (34.1%) | 11 (26.8%) | 16 (39.0%) | 41 |
| **3** | 0 | 25 (18.4%) | 20 (14.7%) | 91 (66.9%) | 136 |
| **Total** | 73 (23.3%) | 84 (26.8%) | 40 (12.8%) | 116 (37.1%) | 313 |

**Abbreviations:** AKI, acute kidney injury

^a^ Duration of AKI was measured from the onset of AKI until the day when serum creatinine returned to <50% of baseline without requirement of kidney replacement therapy. Duration of AKI was defined as either transient (≤ 2 days), sustained (3–6 days) or prolonged (≥ 7 days or non-recovery).
